# Genotoxic carcinogenicity of pyrrolizidine alkaloids: relevance of potency factors for the risk assessment

**DOI:** 10.1007/s00204-025-04182-1

**Published:** 2025-09-19

**Authors:** Benjamin Sachse, Stefanie Hessel-Pras, Bernd Schäfer

**Affiliations:** 1 Department of Food and Feed Safety in the Food Chain, German Federal Institute for Risk Assessment, Max-Dohrn-Straße 8-10, 10589 Berlin, Germany; 2 Department of Chemical and Product Safety, German Federal Institute for Risk Assessment, Max-Dohrn-Straße 8-10, 10589 Berlin, Germany

**Keywords:** Pyrrolizidine alkaloids, Potency, Genotoxicity, Carcinogenicity, Risk assessment

## Abstract

Pyrrolizidine alkaloids represent a large group of substances synthesized as secondary plant metabolites. The subgroup of the 1,2-unsaturated pyrrolizidine alkaloids (PAs) can induce acute and chronic liver toxicity in humans and livestock, and many of them are considered to be genotoxic and carcinogenic. Currently, the risk assessment is based on carcinogenicity data for the relatively potent PA riddelliine, and on the assumption that all PAs, regardless of their chemical structure, have the same potency. However, results from in vitro and in vivo studies indicate that the severity of the effects depends on the alkaloids' structure and, thus, varies for different congeners. To align the concept of potency factors with risk assessment, we analysed the available studies and selected those that best reflect the human situation or mode of action, and that included riddelliine in their study design. Based on the selected studies, we used riddelliine as a reference point to calculate the relative potencies of the PAs also used in the corresponding study. Further, we propose conservatively derived relative potency factors for certain PA congeners and evaluated the impact when applying them to real food samples considering tea and food supplements as examples. Our evaluation demonstrates that the inclusion of relative potency factors for PAs has generally only a minor influence on the final risk assessment, especially when conservative factors are used. However, this approach may be of great importance in certain situations where the PA spectrum consists mainly of PAs with low potency, such as the monoester lycopsamine.

**Supplementary Information:**

The online version contains supplementary material available at 10.1007/s00204-025-04182-1.

## Introduction

Pyrrolizidine alkaloids represent a class of secondary metabolites found in higher plants, mainly in the families Asteraceae, Boraginaceae, and Fabaceae (Smith and Culvenor 1981) and are considered to be part of their chemical defence (Reinhard et al. 2009; Wink 2019). Several hundred congeners have already been identified (Wiedenfeld et al. 2008). Based on chemotaxonomic considerations, pyrrolizidine alkaloids are expected to occur in more than 6000 plant species worldwide (Teuscher and Lindequist 2010).

Chemically, pyrrolizidine alkaloids consist of a necine base as the core structure, to which one or two necic acids are attached via an ester Linkage. Based on the esterification, they can be grouped as monoesters, open-chain diesters, and cyclic diesters. Depending on the core structure, pyrrolizidine alkaloids can be subdivided further into retronecine, heliotridine, otonecine, and platynecine type pyrrolizidine alkaloids. The first three groups have a double bond in the 1,2 position of the necine base, whereas platynecine type pyrrolizidine alkaloids are fully saturated (Hartmann and Witte 1995).

Pyrrolizidine alkaloids containing a double bond in the 1,2 position of the necine base are generally considered to be toxic. The liver is the main target organ affected by the toxicity of 1,2-unsaturated pyrrolizidine alkaloids (PAs). Other organs, particularly the lungs, may also be damaged (Allgaier and Franz 2015; Chen et al. 2010; Edgar et al. 2015; Fu et al. 2004).

**Fig. 1.**
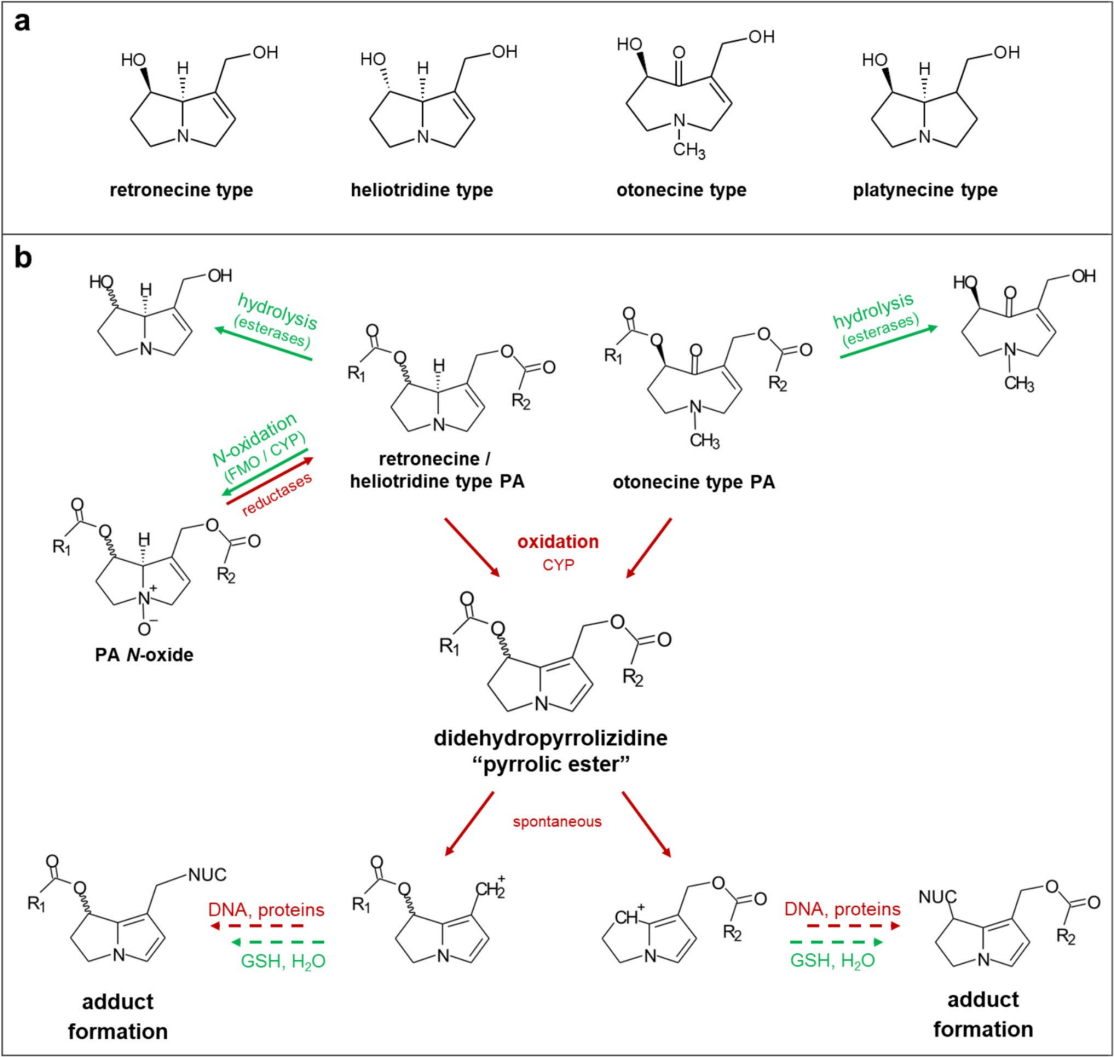
**a** The necine core structure of different types of pyrrolizidine alkaloids. Pyrrolizidine alkaloids with a double bond in the 1,2 position are generally considered to exert toxicity, whereas platynecine types with a saturated ring system are assessed to be non-toxic. **b** Metabolic pathways of 1,2-unsaturated pyrrolizidine alkaloids (PAs) of the retronecine, heliotridine, or otonecine type. The metabolism of PAs involves detoxifying (green) and toxifying (red) reactions. Three major metabolic pathways are described: (1) hydrolysis, which forms the free necine base and acid(s); (2) *N*-oxidation, and (3) oxidation/oxidative *N*-demethylation, which leads to the reactive intermediate didehydropyrrolizidine (pyrrol ester). Didehydropyrrolizidine reacts with nucleophilic centres, for example those found in glutathione (GSH) or water, proteins or DNA. GSH conjugation, *N*-oxidation, and hydrolysis are generally detoxifying reactions, whereas adduct formation with DNA and proteins may provoke toxicity. R, necic acid; CYP, cytochrome P450 monooxygenase; FMO, flavin monooxygenase; NUC, nucleophilic compound; DNA, desoxyribonucleic acid.

**Fig. 2:**
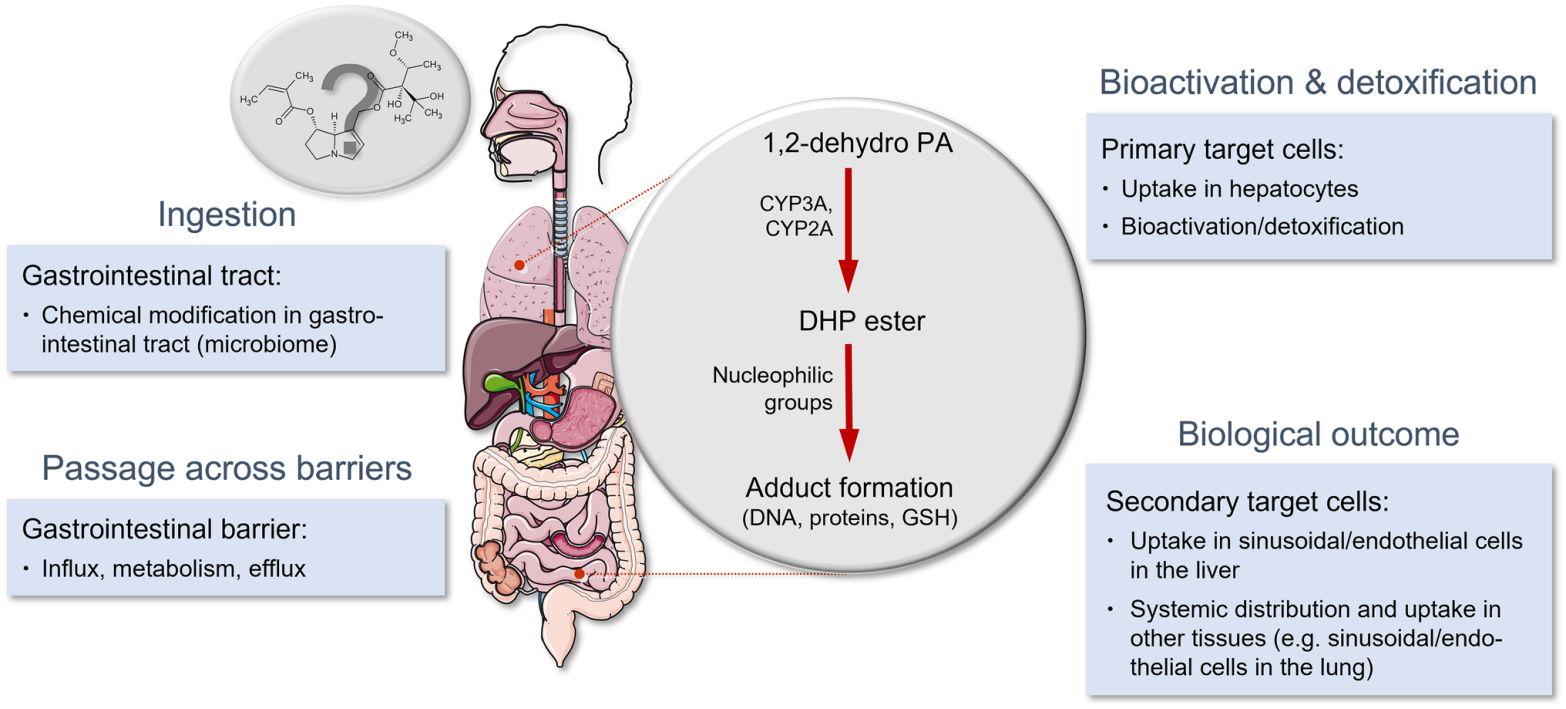
Impact of toxicokinetics and toxicodynamics on the potency of structurally diverse PAs, and the resulting challenge for risk assessment. This figure was created using images from Servier Medical Art Commons Attribution is licensed under the Creative Commons Attribution 4.0 International License (CC BY 4.0), (http://smart.servier.com)

PAs are protoxins that can be bioactivated to highly reactive pyrrolic esters, which are capable of covalently binding to nucleophilic structures such as proteins or DNA. The modifications of these biomolecules can lead to either cytotoxicity or mutations, respectively. These modifications are, therefore, considered to be the primary cause of both, the non-neoplastic lesions occurring in the sinusoidal epithelial liver cells and the genotoxic-carcinogenic effects (Allgaier and Franz 2015; Fu 2017; IPCS/INCHEM 1988; Ruan et al. 2014; Wiedenfeld et al. 2008). In humans, bioactivation appears to be mainly mediated by the cytochrome P450 monooxygenases (CYP) 3A4, 3A5, and 2A6 (Ruan et al. 2014). Metabolism can, however, also lead to detoxification. Such pathways include the hydrolysis of the ester bond by esterases and the *N*-oxidation of the necine base in the case of retronecine and heliotridine type PAs, as well as glutathione conjugation (Allgaier and Franz 2015; Fu et al. 2004; Wiedenfeld et al. 2008). It is worth noting that recent research has shown that, in the case of PAs, glutathione conjugates should still be considered reactive metabolites (Xia et al. 2015) (Fig. 1). Together, the pathways that dominate the metabolism of individual PA congeners are expected to have a significant impact on the relative toxic potency of individual PAs. Next to the metabolic activation in the target cell, further toxicokinetic factors are believed to influence the potency of individual PA congeners. The high degree of uncertainty resulting from the complex toxicokinetics emphasizes the need for additional toxicological data relating to these aspects, such as absorption in the gastrointestinal tract, systemic distribution, uptake in the target cells, and export mechanisms releasing reactive metabolites from the target cells, and excretion, as well as metabolic activation in target cells, and the genotoxic potency of the many individual PAs (Fig. 2). This could then enable a substantial refinement of the risk assessment.

Only a minority of plants synthesizing PAs are commonly used as food. Examples include borage and certain plants used in food supplements. However, the main source of PAs in the food chain is the co-harvesting of PA-containing plants growing on agricultural areas. In addition, bee products can become contaminated with PAs via pollen from PA-containing plants. The PA transfer from contaminated feed to animal products such as milk and meat can occur (Sachse et al. 2020).

Due to their toxic properties, the presence of PAs in food represents a health concern. Severe and sometimes fatal intoxications have occasionally been reported following exposure to very high levels of 1,2-unsaturated PAs, e.g. in Afghanistan. These occurred in the context of highly contaminated grains caused by *Heliotropium* species (Kakar et al. 2010; Molyneux et al. 1991). In addition, cases of intoxication have been reported in Asian regions after ingestion of herbs used in traditional Chinese medicine, as these may either contain 1,2-unsaturated PAs themselves or can be confused/contaminated with other PA-containing plants (Dai et al. 2007; Ma et al. 2018). Hepatic sinusoidal obstruction syndrome is the typical clinical symptom complex of an acute PA poisoning in humans (Allgaier and Franz 2015).

Data from animal studies have further demonstrated a genotoxic and carcinogenic potential of many PAs (Allgaier and Franz 2015; Chen et al. 2010; Edgar et al. 2015; Fu et al. 2004). Animal studies with riddelliine have shown that the DNA adduct level and mutation frequency in liver cells correlate with the carcinogenicity (Chen et al. 2010). The characteristic DNA adducts are, therefore, considered to be the initial step in the formation of PA-induced cancers (Fu 2017). Pyrrole–protein adducts (PPAs) have been observed in the blood of highly exposed humans suffering from PA-induced liver toxicity in Asian regions. These adducts can be considered as biomarkers reflecting the internal exposure to the highly reactive metabolites. The occurrence of PPAs, in turn, demonstrates that the reactive pyrrole esters are indeed formed in humans (Gao et al. 2015; Lin et al. 2011; Ma et al. 2018; Ruan et al. 2015). Consequently, it is assumed that the genotoxic-carcinogenic activity observed in rodents is also relevant to humans (Sachse et al. 2020). In addition, He et al*.* reported that PAs lead to a characteristic mutation signature which was observed in animal experiments and in in vitro studies using HepaRG cells. This signature was also found in liver cancer samples from patients with PA exposure, as indicated by the presence of PPAs, but not in PPA-negative samples. Furthermore, He et al*.* observed that this signature was relatively prevalent in liver cancer samples from Asia but generally absent in liver cancers in Western countries (He et al. 2021b).

Genotoxic carcinogenicity is considered to be the most sensitive endpoint. Consequently, no safe intake level or health-based guidance value can be derived (BfR 2020; EFSA 2017; JECFA 2020). As usual for such compounds, the margin of exposure approach (MOE) is applied in the risk assessment to prioritise the need for risk management measures. The MOE is the ratio of an appropriate toxicological reference point to the estimated human exposure to the substance. Among the PAs, lasiocarpine and riddelliine have the most relevant data on carcinogenicity. The European Food Safety Authority (EFSA) used the formation of haemangiosarcomas observed in a 2-year cancer study in rats orally exposed to riddelliine to derive a BMDL_10_ (lower confidence limit of the dose that is associated with an extra risk of 10% on tumour incidence) of 237 µg/kg body weight (bw) as a toxicological reference point to assess the cancer risk (EFSA 2017). This value is then divided by the estimated human exposure to calculate the MOE (BfR 2020; EFSA 2017).

The estimated human exposure used to calculate the MOE is based on determination of the sum of 21 individual PA congeners plus 16 potentially co-eluting isomers that are routinely analysed in the considered food items (BfR 2020). This approach assumes that all congeners have an equivalent toxic potency. However, there is evidence that individual PAs actually differ in their relative toxicity. In 2016, Merz and Schrenk were the first to systematically evaluate the relative toxic potency of individual PA congeners based on the available literature. They published interim relative potency (iREP) factors for a number of congeners. Using data on acute toxicity in rodents, in vitro cytotoxicity and genotoxicity findings in the fruit fly *Drosophila melanogaster*, they proposed the following potency factors: 1 for cyclic diesters and open-chain diesters with 7*S* configuration; 0.3 for monoesters with 7*S* configuration (heliotridine type); 0.1 for open-chain diesters with 7*R* configuration; and 0.01 for monoesters with 7*R* configuration. The same factors were assigned to the respective *N*-oxides (Merz and Schrenk 2016). In the meantime, further studies—mainly conducted in vitro—have provided additional information on the differences in the relative toxic potencies of individual PAs (e.g. Allemang et al. 2018; Chen et al. 2019; Haas et al. 2023; He et al. 2021a, 2017; Lester et al. 2019; Louisse et al. 2019; Pan et al. 2023)).

To substantiate the discussion on the relative toxic potencies of individual PAs and to draw attention to the expected differences in the (geno)toxic and potentially carcinogenic potency of numerous PAs, we aimed to provide an overview of relevant studies investigating the relative toxic potency of PA congeners. Furthermore, based on the available data, we attempt to identify conservative potency factors and evaluate their implications for risk assessment when applied to real food samples. By analysing the variability of the potency factors of individual PAs depending on the study design used for the derivation, we asked if such potency factors would allow for substantial refinement of the risk assessment. Finally, we discuss options for further improving the database.

### Data base on the relative toxic potency of PA congeners

#### Scientific literature for relevant studies

Over the years there have been numerous studies investigating the relative toxic potency of individual PAs (e.g. Allemang et al. 2018; Chen et al. 2019; Chou et al. 2003; Haas et al. 2023; He et al. 2021a, 2017; Lester et al. 2019; Louisse et al. 2019; Merz and Schrenk 2016; Pan et al. 2023; Xia et al. 2006, 2004, 2008, 2016, 2013). These have used many different assays, comprising in vitro and in vivo approaches. The former includes, *inter alia,* the micronucleus test, the γH2AX assay, the comet assay, studies on DNA and protein adduct formation, and various cytotoxicity tests; the latter include, *inter alia,* studies on DNA and protein adduct formation and studies on lethal doses.

In the context of the present work, we have searched the scientific literature for relevant studies. Here, we will present an overview of the results. The main aim was to get an impression of the comparability and consistency of the potency factors that result from different studies—considering similar but also different endpoints. Studies were searched in *Scopus* using the search string "pyrrolizidine alkaloid*"AND potency* within *article title, abstract, and key words*. All studies found are shown in the supplementary information (Table S1). Relevant studies were identified by applying a number of inclusion criteria.

#### Criteria for the inclusion or exclusion of data

Studies were only considered if an appropriate dose–response metric, such as a benchmark dose or EC_50_, was reported. An exception was made, however, for studies on adduct formation, as these were considered highly relevant, despite generally being conducted at only one to three dose levels. In these cases, the results of the individual dose groups were compared quantitatively. If more than one dose level was tested in a study, the results for each dose group were considered as an individual data set and compared individually. In studies, in which both a benchmark dose and a benchmark dose lower confidence limit were reported as dose–response metric, the benchmark dose was used for potency comparison.

Studies were only taken into account if all relevant information, e.g. dose/test concentration, incubation time in in vivo studies, were clearly described. Furthermore, studies using model systems that do not appropriately reflect the metabolism of PAs were excluded from further evaluation. This includes studies using cell lines overexpressing human CYP3A4. While these cell lines are useful for mechanistic studies, the results obtained cannot be used to draw quantitative conclusions, as the metabolism is heavily biased towards activating transformations, with detoxifying reactions being underrepresented.

In recent risk assessments of pyrrolizidine alkaloids, the toxicological reference point used in the MOE approach was derived from carcinogenicity findings observed in a rat study with riddelliine as described in the introduction. Therefore, in the present evaluation studies were considered only if riddelliine was included in the data set to allow for a normalization of the other congeners.

Some of the available studies have only presented their results as graphical figure, with no numerical values available. In these cases, the individual potency factors have been estimated and are presented as numbers in the tables of the supplementary information. However, these numbers have not been included in further quantitative evaluations.

The cytotoxicity of PAs has been studied at different time points. However, only the cytotoxicity at 24 h has been included in further quantitative evaluations. If other tests were conducted at different time points, the results of different time points were considered as individual data sets. The supplementary information provides a rationale for each study that was not included in the further quantitative assessment (Table S1).

This search strategy yielded 16 publications and a total of 28 relevant data sets, which could be used for quantitative evaluations (see supplementary information, Table S1). In cases where the strength of the effect has been too weak to determine, i.e. values below a limit of quantification, or cytotoxicity too weak to calculate an EC_50_, etc., these measurements have been set to a relative potency of 0.

#### Criteria for grouping the different studies

To evaluate the relative toxic potency of individual PA congeners, the relevant data were grouped according to different endpoints to check for the consistency of potency factors:

- Genotoxicity in vitro—human liver cells (micronucleus test in HepaRG cells, γH2AX assay in HepaRG cells or primary human hepatocytes)
- Genotoxicity in vitro—rat liver cells (γH2AX assay in primary rat hepatocytes)
- Adduct formation in a cell-free system (DNA adducts after incubation with rat liver microsomal activation)
- Adduct formation in vitro—rat liver cells
- Cytotoxicity in vitro—human liver cells
- Adduct formation in rats dosed p.o. (DNA adducts, protein adducts)
- Protein adducts in the liver of zebra fish dosed p.o.
- LD_50_ in mice after *intra venous* (i.v.) application
- LD_50_ in adult rats after *intra peritoneal* (i.p.) application

Groups c and d were separated from groups a and b because the adduct data represent single-dose measurements, and thus, are not fully comparable with the data from groups a and b that represent completely modelled dose–response information. For groups for which more than one data set was available, the data are presented in Tables S3 to S6 in the supplementary information.

### Conservative relative toxic potencies of PAs derived from the different studies

The effect sizes of individual PAs within each single data set were normalized to the effect size of concomitantly studied riddelliine. This yielded a relative potency factor for all investigated PAs within one data set in comparison to riddelliine which was used as the reference. At that level, potency factors were only calculated within the individual data sets—see Tables in the supplementary information.

If more than one data set was available in a group, for each individual PA, a group mean was calculated. Of note, the basis for the group mean is highly variable, i.e. the number of data points available that could be considered for the group mean value varies substantially between PA congeners (between 1 and 8)—see Tables S3 to S6 in the supplementary information.

The resulting group mean values, presented in Table 1, provide a rough overview on the consistency of potency factors that are derived for the different endpoint groups. The data show that the mean group potency factors are in many cases roughly in a similar range (Table 1).

**Table 1:** Relative toxic potencies for different endpoints compared to riddelliine

PA	Group a: Genotoxicity in vitro – human liver cells	Group b: Genotoxicity in vitro – rat liver cells	Group c: Adduct formation in a cell-free system	Group d: Adduct formation in vitro – rat liver cells	Group e: Cytotoxicity in vitro – human liver cells	Group f: Adduct formation in rats dosed p.o	Group g: Protein adducts in the liver of zebra fish dosed p.o	Group h: LD_50_ in mice after i.v. application	Group i: LD_50_ in rats after i.p. application	iREP proposed by Merz and Schrenk (2016)
*Open-chain diester PA*										
Heliosupine (7*S*)	0.7								2	1
Lasiocarpine (7*S*)	4	4	2	1	6	1	2	1	1	1
Lasiocarpine-NO (7S)	0.004		0.7	0.1					1	1
7-Acetylintermedine (7*R*)	0.7									
7-Acetyllycopsamine (7*R*)	0.6									
Echimidine (7*R*)	0.6			0.6	0#				0.5	0.1
Symphytine (7*R*)									0.8	0.1
*Monocyclic diester PA*										
Epi-Jacobine (7*R*)	0.5									
Erucifoline (7*R*)	0.3									
Integerrimine (7*R*)	0.8			0.3						
Jacobine (7*R*)	1									
Jaconine (7*R*)	0.8									
Jacoline (7*R*)	0.2									
Merepoxine (7*R*)	0.7									
Mereskine (7*R*)	0.9									
Monocrotaline (7*R*)	0.2		0.4	0.6	0#	1	0.4		1	1
Monocrotaline-NO (7*R*)			0.07	0.1						1
Retrorsine (7*R*)	3		0.9	1	3	2	2	2	3	1
Retrorsine-NO (7*R*)	0#		0.3	0.2			0.3	0.1	0.4	1
Riddelliine (7***R***)	1	1	1	1	1	1	1	1	1	1
Riddelliine-NO (7*R*)	0.01		0.3	0.2		0.5	0.3			1
Senecionine (7*R*)	1			0.4	0#			2	2	1
Senecionine-NO (7*R*)	0#									1
Seneciphylline (7*R*)	1			0.3	0#			1	1	1
Senecivernine (7*R*)	1									
Trichodesmin (7*R*)	0.1									
Usaramine (7*R*)	0.2									
Clivorine (O)			0.7	0.4		0.2	0.7			
Otosenine (O)	0.1									
Senkirkine (O)	0.2			0.2		0.6			0.5	1
Platyphylline (P)	0.03		0#	0#		0#	0#			
*Monoester PA*										
Heliotrine (7*S*)	0.7		0.3	0.5	0#	0.3	0.2	0.4	0.4	0.3
Heliotrine-NO (7*S*)	0.005		0.1	0.08						
Echinatine (7*S*)	0#								0.3	0.3
Europine (7*S*)	0.1			0.3	0#					
Rinderine (7*S*)	0#									
Indicine (7*R*)	0#			0.1	0#				0#	0.01
Intermedine (7*R*)	0.01			0.2					0.07	0.01
Lycopsamine (7*R*)	0.04			0.1	0#	0.1				0.01
Trachelamzhamine (P)	0#									
*Non-ester PA*										
Heliotridine (7*S*)	0#									
Retronecine (7*R*)	0#					0.08				
Retronecine-NO (7*R*)	0#									
Otonecine (O)		0								

The effect sizes of individual PAs within each data set were normalized to the effect size of the concomitantly studied riddelliine resulting in a relative potency factor for all PAs investigated within this one data set. If more than one dataset was available for a group of PAs, a group mean was calculated. The data here represent the group mean values of all the data considered within a group. In addition, interim relative potency (iREP) factors as proposed by Merz and Schrenk (2016) are shown for comparison

^a^7*S*, heliotridine type; 7*R*, retronecine type; O, otonecine type; P, platyphylline type; #, values are solely based on data, where effects were too weak for determination and measurements have been set to a relative potency of 0

Since reactive pyrrole metabolites, which are thought to be responsible for the toxic effects, should affect all endpoints represented in the groups and the available data did not so far support to prefer the data from one group over the data from another group, we decided to use the individual relative potencies from all relevant data sets from all groups to further evaluate the overall fluctuation in the available data (Table 7 in the supplementary information). We calculated the overall mean relative potency as well as standard deviation and relative standard deviation for each single PA congener. The highest variability in the underlying data, as reflected by a relative standard deviation of 100% or more, was observed for lasiocarpine* N*-oxide, indicine, lycopsamine, and echinatine as well as the fully saturated platyphylline and the non-ester PA retronecine. Of note, the underlying data basis is highly variable, ranging from 1 to 23 data points per individual congener. There was a notable tendency for the relative toxic potency of open-chain and cyclic diesters to be higher than that of most monoesters (Table 2).

**Table 2:** Overall toxic potency of individual PAs relative to riddelliine

PA	Mean	Standard deviation	Relative standard deviation (%)	Minimum	Maximum	No of data points	Proposed iREP—min	Proposed iREP—mean	Proposed iREP—max	iREP proposed by Merz and Schrenk (2016)
*Open-chain diester PA*										
Heliosupine (7*S*)	1	1	45	0.7	2	2	1	1	1	1
Lasiocarpine (7*S*)	2	2	97	0#	9	23	0	1	1	1
Lasiocarpine-NO (7S)	0.2	0.2	104	0#	0.7	11	0*	1*	1*	1
7-Acetylintermedine (7*R*)	0.7			0.7	0.7	1	1	1	1	
7-Acetyllycopsamine (7*R*)	0.6			0.6	0.6	1	1	1	1	
Echimidine (7*R*)	0.5	0.5	83	0#	2	12	0	1	1	0.1
Symphytine (7*R*)	0.8			0.8	0.8	1	1	1	1	0.1
*Monocyclic diester PA*										
Epi-Jacobine (7*R*)	0.5			0.5	0.5	1	1	1	1	
Erucifoline (7*R*)	0.3			0.3	0.3	1	1	1	1	
Integerrimine (7*R*)	0.4	0.2	53	0.1	0.8	8	0.1	1	1	
Jacobine (7*R*)	1			1	1	1	1	1	1	
Jaconine (7*R*)	0.8			0.8	0.8	1	1	1	1	
Jacoline (7*R*)	0.2			0.2	0.2	1	1	1	1	
Merepoxine (7*R*)	0.7			0.7	0.7	1	1	1	1	
Mereskine (7*R*)	0.9			0.9	0.9	1	1	1	1	
Monocrotaline (7*R*)	0.7	0.5	75	0#	2	20	0	1	1	1
Monocrotaline-NO (7*R*)	0.09	0.04	44	0.002	0.1	9	0*	1*	1*	1
Retrorsine (7*R*)	2	2	95	0.3	9	18	1	1	1	1
Retrorsine-NO (7*R*)	0.2	0.1	53	0#	0.4	13	1*	1*	1*	1
Riddelliine (7***R*)**	1	0	0	1	1	28	1	1	1	1
Riddelliine-NO (7*R*)	0.3	0.2	71	0#	0.8	17	1*	1*	1*	1
Senecionine (7*R*)	0.7	0.6	86	0#	2	11	0	1	1	1
Senecionine-NO (7*R*)	0#			0#	0#	1	0*	1*	1*	1
Seneciphylline (7*R*)	0.5	0.5	91	0#	1	11	0	1	1	1
Senecivernine (7*R*)	1			1	1	1	1	1	1	
Trichodesmin (7*R*)	0.1			0.1	0.1	1	0.1	0.1	0.1	
Usaramine (7*R*)	0.2			0.2	0.2	1	1	1	1	
Clivorine (O)	0.4	0.2	56	0#	0.7	9	0	1	1	
Otosenine (O)	0.1			0.1	0.1	1	0.1	0.1	0.1	
Senkirkine (O)	0.4	0.3	67	0.06	1	13	0.1	1	1	1
Platyphylline (P)	0.002	0.01	374	0#	0.03	15	0	0.01	0.1	
*Monoester PA*										
Heliotrine (7*S*)	0.4	0.3	74	0#	1	21	0	1	1	0.3
Heliotrine-NO (7*S*)	0.08	0.06	73	0.005	0.2	9	0*	1*	1*	
Echinatine (7*S*)	0.2	0.2	100	0#	0.3	2	0	1	1	0.3
Europine (7*S*)	0.1	0.1	72	0#	0.3	4	0	0.1	1	
Rinderine (7*S*)	0#			0#	0#	1	0	0	0	
Indicine (7*R*)	0.03	0.04	138	0#	0.1	5	0	0.1	0.1	0.01
Intermedine (7*R*)	0.09	0.08	88	0#	0.2	5	0	0.1	1	0.01
Lycopsamine (7*R*)	0.1	0.1	113	0#	0.5	16	0	0.1	1	0.01
Trachelamzhamine (P)	0#			0#	0#	1	0	0	0	
*Non-ester PA*										
Heliotridine (7*S*)	0#			0#	0#	1	0	0	0	
Retronecine (7*R*)	0.07	0.10	147	0#	0.2	5	0	0.1	1	
Retronecine-NO (7*R*)	0#			0#	0#	1	0	0.1*	1*	
Otonecine (O)	0#			0#	0#	1	0	0	0	

This table illustrates the overall mean values that resulted from all available data together with further statistical aspects. Furthermore, interim relative potency (iREP) factors are proposed and compared with iREP factors that have been proposed by Merz and Schrenk (2016).

7*S*, heliotridine type; 7*R*, retronecine type; O, otonecine type; P, platyphylline type; *, for NO´s the values of the respective parent PA was used instead of the measured values; #, values are solely based on data, where effects were too weak for determination and measurements have been set to a relative potency of 0.

#### Discussion on the informative value of the derived relative potency factors for PAs

The relatively large scatter in relative potency factors that are derived using different assays suggests low reliability.

Nevertheless, we were interested in the magnitude of potential overall potency factors when conservatively derived based on the available data. We used the overall mean, as well as the overall minimum and maximum, of the available data (Table 2) as a basis. Because of the relatively pronounced scatter, potency differences were only taken into account if the difference was at least one order of magnitude. Thus, PA congeners with values within one order of magnitude relative to riddelliine were given a relative potency of 1, PAs with values ≤ 0.1 or ≥ 10 were set to 0.1 or 10, etc.

When using the overall mean as a basis, most of the PA congeners were given a potency factor of 1. A lower potency factor of 0.1 was observed for the macrocyclic diesters trichodesmine and otosenine, as well as the monoesters europine, indicine, intermedine, and lycopsamine. The only available data points for the monoesters rinderine and trachelamzhamine were below the limit of detection and were, therefore, set to 0. When using the overall maximum of the available data as a basis, even more congeners were given a potency factor of 1. Only for trichodesmine, otosenine, and indicine a lower value of 0.1 was derived. Again, rinderine and trachelamzhamine were set to 0. In comparison, much lower relative potencies were observed when considering the overall minimum of the available data as a basis. In this scenario, several derivatives were given a relative potency factor of 0.1 (integerrimine, trichodesmine, otosenine, senkirkine), or were even set to 0 (lasiocarpine!!!, echimidine, monocrotaline, senecionine, seneciphylline, clivorine, heliotrine, echinatine, europine, rinderine, indicine, intermedine, lycopsamine, trachelamzhamine). However, using the overall minimum values appears inappropriate, since even PAs with a well-accepted high potency were set to 0. This might be a result of individual, inappropriate test results. Therefore, calculations that are based on the overall mean appear to be most suitable.

The relative potencies for the Fully saturated congener platyphylline and the 1,2-unsaturated but non-esterified derivatives were generally low (Table 2).

PA *N*-oxides generally showed a (much) lower potency than their respective parent compounds, especially in some in vitro assays (Table 1 and Tables in the supplementary information). However, it is known that PA *N*-oxides can be almost completely converted to PAs by bacterial reductases in the gut and also by cellular reductases in the liver (Allgaier and Franz 2015; Yang et al. 2019, 2017). Recently published studies indicate that the relative potency of PA *N*-oxides is almost as high as that of the parent PAs, particularly at low intake levels (Widjaja et al. 2023a, 2022, 2023b). At least in Western countries, low exposure levels can be assumed for humans. Therefore, it appears appropriate to apply the potency factor of the parent PAs also for the respective *N*-oxides.

Of note, the relative potency factors derived via our approach are generally less pronounced than potency factors that have been suggested by other authors—at least when considering the overall mean or maximum values. There are several reasons for this discrepancy. First, we derived the potency factors in an individual data set by comparing the effect sizes of individual PAs with riddelliine – not with the most potent PA in the respective data set. Second, we derived the mean relative potency factors based on the result of several in vitro and in vivo assays that may be assumed to reflect the formation of the toxic pyrrol esters.

### Consequences for the risk assessment

As a next step, we wanted to evaluate how the consideration of potency factors would affect the risk assessment of foods. To this end, we compared PA levels in (herbal) teas and in food supplements as examples, that were calculated by considering either the equal potency of individual PAs or the overall mean relative potency factors depicted in Table 2.

The PA levels were taken from a publication by Mulder et al*.* (2015). We evaluated the impact for different types of tea separately, namely green tea, black tea, peppermint tea, chamomile tea, mixed herbal tea, and rooibos tea. Food supplements were divided into three categories: plant extract formula, pollen-based supplements, and other dietary supplements. We evaluated the impact by comparing the overall mean relative potency factors derived above in comparison to the currently applied approach of considering each measured PA as equally potent.

Based on the data set examined for tea, the highest impact of considering potency factors was observed for black tea and green tea. In this case, the consideration of relative potency factors led to a decrease of about 70% and 45%, respectively. In contrast, the impact was minute for rooibos tea (~ 5%), peppermint tea (~ 5%), mixed herbal tea (~ 15%), and chamomile tea (~ 20%). For the food supplement samples, there was a reduction of approx. 25%, 45%, and 60% for pollen-based supplements, plant extract formula, and other dietary supplements, respectively (Supplementary information Table S14).

Chen et al. ( 2022) also evaluated the impact of considering relative potency factors on the risk assessment for herbal teas. The authors applied the interim relative potency factors derived by Merz and Schrenk in 2016 and evaluated 21 types of (herbal) teas. As in our analyses, the authors observed a highly variable impact depending on the type of tea. In their study, the impact was negligible for green tea, rooibos, tephroseris, Gynura segetum, lemon balm, and mixed herbs sample 1. It was small for chamomile (~ 10%), eupatorium (~ 10%), asteraceae (~ 20%), heliotropium (~ 30%), and mixed herbs sample 2 (~ 30%). The reduction was quite pronounced for earl grey (~ 75%), sage & lemon myrtle (~ 75%), forest fruit (70%), lemon balm & liquorice (~ 70%), lemon verbena (~ 70%), and mixed herbs sample 3 (~ 90%). The highest impact was observed for lungwort (~ 98%) and borage (> 99%). Although these results are not directly comparable with our study, e.g. due to the consideration of other potency factors and a larger number of PAs considered, the findings emphasize that the impact is predominantly affected by the individual PA profile.

It can be concluded that in many cases the inclusion of relative potency factors for PAs will only modestly impact the final risk assessment, especially when conservatively derived factors are used. However, it may be highly relevant in certain situations, where the PA content consists mainly of low potency PAs, such as the monoester lycopsamine.

### Uncertainties associated with the risk assessment of pyrrolizidine alkaloids

The risk assessment of PAs is subject to several uncertainties, relating to all levels of the assessment. As mentioned above, several hundred PAs have already been identified. However, only a limited number of 1,2-unsaturated PAs is currently routinely determined in food samples. According to current recommendations, 21 PA congeners plus 16 potentially co-eluting isomers are included in standard analytical methods. Although the available data suggest that these procedure covers the majority of the total PA content of a sample in many cases (BfR 2020), it may lead to a relevant underestimation of the actual PA levels in certain food samples. For example, some PA congeners that have been observed to predominantly pass over to animal-based products like milk and meat, are not currently covered by the commonly used analytical method (Taenzer et al. 2025).

Furthermore, with regard to the exposure assessment, it is important to mention that the data base for certain food categories, such as herbs and spices, is quite insufficient. This is due to limited data on occurrence levels in these food categories, but also limited data on consumption levels. In turn, this will result in some degree of underestimation of human exposure (BfR 2020).

Beside the uncertainties that are related to the overall human exposure, several uncertainties are related to the hazard characterisation of PAs. Next to potential differences in the relative potency of individual congeners, these uncertainties relate to the hazard characterisation of PAs in general. For example, the BMDL_10_ of 273 µg/kg bw derived on the basis of dose–response data on the formation of haemangiosarcomas in a 2-year cancer study in rats represents the most suitable toxicological reference point for assessing the genotoxic-carcinogenic risks of PAs (BfR 2020). However, at the same time, it is well known that higher doses of PAs—as have been used in the carcinogenicity study – also lead to concurrent cytotoxicity and chronic inflammation (Merz and Schrenk 2016). This may, in turn, promote cancer formation in the animal studies, thereby limiting the quantitative interpretation of the resulting dose–response relationship with respect to low-dose exposure scenarios. Therefore, such high-dose effects may result in a significant overestimation of the actual risk in humans.

The consideration of potency factors as they have been suggested above, may lead to a slightly more realistic assessment with respect to potency differences of individual PA congeners. However, in view on the existing overall uncertainties, the small impact appears to be of low relevance and may create the impression of an accuracy that does not exist in reality. Use of potency factors that have been derived by others and suggest higher differences between individual PA congeners would further increase such uncertainty.

In addition, relative potency factors that are mainly derived on the basis of in vitro data have some limitations. These are mainly due to the fact that toxicokinetics—in particular metabolic activation/inactivation in combination with transport processes—cannot be fully captured in its complexity in these tests. In turn, the integration of such potency factors in the risk assessment would add some level of additional uncertainty at the current stage. Sophisticated in vitro to in vivo extrapolation (IVIVE) models may help to overcome these limitations in future.

### What is needed to better account for differences in PA potency?

From the risk assessor´s perspective, a current integration of conservatively derived potency factors would not significantly increase the relevance of human risk assessments on the overall exposure to PAs. This is mainly due to the uncertainties concerning all levels of the risk assessment, including additional uncertainties that are associated with the use of potency factors.

To better account for inherent differences in the potency of individual congeners, the data basis would need to be further enhanced. Traditionally, relevant data can be obtained in animal studies evaluating relevant endpoints quantitatively, since toxicokinetics is fully covered in vivo. For the sake of animal welfare, this may also be reached by a battery of suitable and relevant in vitro tests. However, at the moment, there are many in vitro studies in which various PA congeners have been investigated in a variety of in vitro models for mechanistic studies on the one hand and for potency evaluation on the other, but only a few could be included in our analysis due to inherent limitations. In future, studies might be more informative if they are a) conducted in a human-relevant cell system, b) focus on human-relevant toxicity endpoints, and c) include riddelliine as a reference compound. Furthermore, it is highly important to integrate mechanistical information on how toxicokinetic aspects, e.g. biotransformation in the gastrointestinal tract, metabolism, and cellular import and export mechanisms, affect the relative potency. Independent of the exact experimental procedure, such a scientific approach would involve a great deal of effort and expense.

Since the latter risk assessments demonstrated that the estimated overall exposure to PAs results in MOE levels above 10,000—suggesting a low concern from a public health point of view—it may be critically questioned if a slight improvement that could be reached justifies effort and costs.

On the other hand, considering conservatively derived potency factors may already be appropriate under certain circumstances—even if such approach adds some new uncertainties. Such situations include the assessment of certain food groups, for which it is known that the PA profile mainly consists of PA congeners with a low potency. If the consideration leads to a drastic difference in the assessment outcome, the additional uncertainties may be of minor relevance.

## Conclusion

In theory, taking into account the relative toxic potency of individual PAs would enable a more realistic risk assessment. However, as shown above, the application of potency factors conservatively derived from the available data would often only have a relatively small impact on the risk assessment outcome, e.g. when considering the exposure via herbal teas. Therefore, a more sophisticated evaluation of the true potency of individual PAs would be needed, which might reveal higher differences in the relative potency. In addition, conservatively derived relative potency factors may already be appropriate for certain food groups, for which it is known that the PA profile mainly consists of PA congeners with a very low potency.

## Supplementary Information

Below is the link to the electronic supplementary material.

Supplementary file1 (XLSX 433 kb)
